# SH2-PLA: a sensitive in-solution approach for quantification of modular domain binding by proximity ligation and real-time PCR

**DOI:** 10.1186/s12896-015-0169-1

**Published:** 2015-06-26

**Authors:** Christopher M. Thompson, Lee R. Bloom, Mari Ogiue-Ikeda, Kazuya Machida

**Affiliations:** Raymond and Beverly Sackler Laboratory of Genetics and Molecular Medicine, Genetics and Genome Sciences, University of Connecticut School of Medicine, 400 Farmington Avenue, 06030 Farmington, CT USA

**Keywords:** SH2, Proximity ligation, SH2-PLA, Modular protein domains, EGFR signaling

## Abstract

**Background:**

There is a great interest in studying phosphotyrosine dependent protein-protein interactions in tyrosine kinase pathways that play a critical role in many aspects of cellular function. We previously established SH2 profiling, a phosphoproteomic approach based on membrane binding assays that utilizes purified Src Homology 2 (SH2) domains as a molecular tool to profile the global tyrosine phosphorylation state of cells. However, in order to use this method to investigate SH2 binding sites on a specific target in cell lysate, additional procedures such as pull-down or immunoprecipitation which consume large amounts of sample are required.

**Results:**

We have developed PLA-SH2, an alternative in-solution modular domain binding assay that takes advantage of Proximity Ligation Assay and real-time PCR. The SH2-PLA assay utilizes oligonucleotide-conjugated anti-GST and anti-EGFR antibodies recognizing a GST-SH2 probe and cellular EGFR, respectively. If the GST-SH2 and EGFR are in close proximity as a result of SH2-phosphotyrosine interactions, the two oligonucleotides are brought within a suitable distance for ligation to occur, allowing for efficient complex amplification via real-time PCR. The assay detected signal across at least 3 orders of magnitude of lysate input with a linear range spanning 1–2 orders and a low femtomole limit of detection for EGFR phosphotyrosine. SH2 binding kinetics determined by PLA-SH2 showed good agreement with established far-Western analyses for A431 and Cos1 cells stimulated with EGF at various times and doses. Further, we showed that PLA-SH2 can survey lung cancer tissues using 1 μl lysate without requiring phospho-enrichment.

**Conclusions:**

We showed for the first time that interactions between SH2 domain probes and EGFR in cell lysate can be determined in a microliter-scale assay using SH2-PLA. The obvious benefit of this method is that the low sample requirement allows detection of SH2 binding in samples which are difficult to analyze using traditional protein interaction assays. This feature along with short assay runtime makes this method a useful platform for the development of high throughput assays to determine modular domain–ligand interactions which could have wide-ranging applications in both basic and translational cancer research.

**Electronic supplementary material:**

The online version of this article (doi:10.1186/s12896-015-0169-1) contains supplementary material, which is available to authorized users.

## Background

An important consequence of reversible phosphorylation of tyrosine residues on proteins is the creation of binding sites for phosphotyrosine recognizing domains such as Src homology 2 (SH2) and phosphotyrosine binding (PTB) domains [1, 2]. The human proteome contains 120 SH2 domains in 110 distinct proteins, and protein-protein interactions mediated by SH2 domains play a critical role in essential cellular processes such as cell growth, migration, differentiation, and survival [3, 4]. We and others have utilized SH2 domains as a tool to profile the global tyrosine phosphorylation state of cells [5–9]. SH2 profiling is a unique proteomic method in which interactions between an array of SH2 domains and protein samples are quantitatively analyzed, thereby defining the functional output of tyrosine phosphorylation. There are three assay platforms for SH2 profiling: quantitative far-Western, rosette, and oligonucleotide-tagged multiplex (OTM) assays [10]. In far-Western, protein samples are separated by electrophoresis and replicate blots are separately probed with labeled SH2 domains [6, 9]. In rosette assay, samples are spotted on a membrane, and the binding assay is carried out in a 96-well plate [7]. The labeled SH2 domain probes are incubated with multiple sample spots in a noncompetitive manner (single SH2 per well). In an oligonucleotide-tagged multiplex (OTM) assay, a mixture of SH2 domains with domain-specific DNA tags are incubated with a sample spot allowing for competitive binding (single sample per well) [8]. Signal is detected either by chemiluminescence (far-Western and rosette) or by PCR (OTM). Quantified values are used to classify samples, such as different cancer tissues, based on SH2 binding preferences (SH2 profiles) [5, 6, 9, 10].

One limitation of current SH2 profiling methods is that these assays themselves do not provide the identity of the binding proteins. When the sample identity is known, such as synthesized peptides or purified proteins, this is not a problem. However, for samples with more complexity such as cell lysate and tissue, it is often difficult to infer the identity of detected SH2 binding proteins unless a literature/database search can provide a helpful clue. In this case, additional experimental methods such as antibody-based or mass spectrometry-based protein identification must be combined with SH2 profiling. For example, in order to identify a band detected by SH2-far-Western, a pull down assay combined with Western blotting with specific antibodies (for few candidate proteins) or MS identification (for an unknown set of proteins) is needed [5, 7]. Further, those additional assays often require a large amount of sample, are labor intensive and not always successful [11]. These laborious, costly procedures are required for even a simple task such as validating the binding of SH2 domains to a few candidate proteins (*e.g.*, EGFR, ErbB2, *etc.*) which have been inferred from SH2 profiling.

Proximity ligation assay (PLA) is a molecular recognition assay that depends on the ligation of two oligonucleotide-tagged probes that are capable of forming a complex, when binding to a target (or targets) that are in close proximity to each other [12, 13]. The method was originally described using aptamers as affinity reagents [14], and in its current version is performed using antibodies to determine protein expression and interactions *in situ* or in solution [15–18]. Various commercial PLA kits are available mainly from Olink Bioscience and Applied Biosystems. Of these, Applied Biosystems TaqMan Protein Assays are designed to quantify target protein expression levels using a small amount of cell lysate (2–3 μl) [19, 20]. The system uses TaqMan technology, an established platform for quantification of gene expression based on quantitative real-time PCR. The standard TaqMan Protein Assay kit was originally designed to detect stem cell markers, while an open kit is available for use with custom antibodies [20]. Although homogenous PLA has been used to determine protein expression in lysate [21, 22], we hypothesized that the system could be customized for detection of SH2 domain-based protein-protein interactions in solution. A PLA-based SH2 profiling method would have multiple advantages such as low sample requirement, higher sensitivity, and rapid validation of SH2 binding protein identity. Here, using activated epidermal growth factor receptor (EGFR) as the SH2 binding target, we have developed and validated such an assay, termed SH2-PLA, which has a broad range of detection, performance equivalent to far-Western, and potential application in translational research [23–25].

## Results

### SH2-PLA assay scheme

**Fig. 1:**
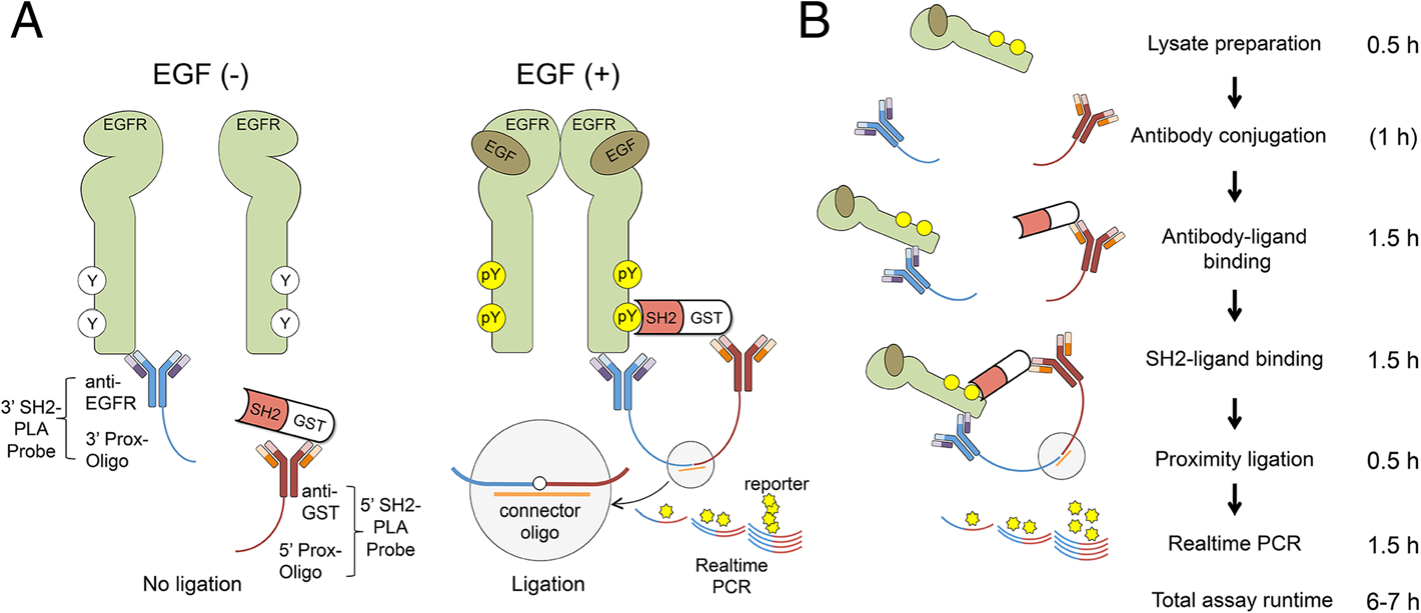
In-solution SH2 domain binding assay using proximity ligation and real-time PCR. **a** *,* Schematic Illustration of SH2-PLA. A pair of PLA probes is used to detect the interaction of tyrosine phosphorylated EGFR and a GST-SH2 domain. The 3′ SH2-PLA probe consists of an anti-EGFR antibody conjugated with the 3′ proximity oligonucleotide (3′ Prox-Oligo). The 5′ SH2-PLA probes consists of an anti-GST antibody conjugated with the 5′ Prox-Oligo and a GST-SH2 domain. When the GST-SH2 domain binds to tyrosine phosphorylation sites of EGFR, the 5′ and 3′ PLA probes are brought in close proximity, allowing ligation of the two Prox-Oligos which is detectable by real-time PCR. **b** *,* Experimental workflow of SH2-PLA Method 1. Lysates are prepared with or without EGF stimulation. Biotinylated anti-GST and anti-EGFR antibodies are conjugated with the 5′ and 3′ Prox-Oligos, respectively, and stored at −20 °C. The 5′ SH2-PLA probe is mixed with purified GST-SH2, and the 3′ SH2-PLA probe is mixed with cell lysates allowing the antibodies to bind their respective epitopes. Subsequently, the 5′ and 3′ PLA probe solutions are combined to induce interaction between the SH2 and pEGFR. Then, the amount of the complex is quantified by proximity ligation and real-time PCR. An alternative method is also possible (Additional file 1: Figure S2). Estimated assay runtime including sample-handling steps for each procedure is noted on the right

We chose the epidermoid carcinoma cell line A431, which overexpresses wild type epidermal growth factor receptor (EGFR), as a developmental platform. The premise of the SH2-PLA assay is that 1) EGF stimulation induces tyrosine phosphorylation of the intracellular domain of EGFR, which creates specific binding sites for SH2 domains such as Grb2, Src, PLCγ1, Vav2, *etc.*; 2) GST-SH2 domain coupled to anti-GST 5′ Prox-Oligo binds to these phosphotyrosines in cell lysate; 3) This interaction brings the anti-GST 5′ Prox-Oligo and anti-EGFR 3′ Prox-Oligo probes together in close proximity, thus allowing for the proximity ligation reaction to be subsequently quantified by real-time PCR (Fig. 1). In other words, detection of a specific assay signal requires the creation of the quaternary complex: anti-EGFR 3′Prox-Oligo probe:phosphorylated EGFR:GST-SH2 protein:anti-GST 5′ Prox-Oligo probe.

### Performance of TaqMan protein expression assay

Prior to customizing the TaqMan Protein Expression Assay for SH2-PLA (*i.e.*, modular domain binding assay), we tested the performance of the original proximity ligation assay using the kit-supplied anti-ICAM1 assay probes and Raji B-cell lymphoma lysate. The 3′ and 5′ oligonucleotide-conjugated anti-ICAM1 assay probes were incubated with the Raji lysate for 60 min followed by the ligation reaction. After heat inactivation, the ligation product was quantified using the TaqMan real-time PCR system. The experiment was performed in a 96 well plate with approximately three hours of assay time. We observed, with high-precision, approximately three orders of magnitude of linear dynamic range for a cell input of 0.24 – 250 cells per μl (intra-assay %CV < 1.1 %) (Additional file 1: Figure S1A). When two independent experiments were compared, they showed very strong correlation (Pearson correlation *r* = 0.99) (Additional file 1: Figure S1B). We concluded that the performance of the TaqMan Protein Expression Assay was sufficient for development of an SH2 domain binding assay.

### Development of the SH2-PLA assay

Biotinylated anti-GST and anti-EGFR polyclonal antibodies were conjugated with 5′ and 3′ oligonucleotides following the probe development protocol (see Methods). To evaluate the anti-GST probes (5′ Prox-Oligo- and 3′ Prox-Oligo-conjugated anti-GST antibodies) and anti-EGFR probes (5′ Prox-Oligo- and 3′ Prox-Oligo-conjugated anti-EGFR antibodies), purified GST protein and A431 cell lysate were serially diluted and respective TaqMan protein expression assays were performed as in the ICAM1 expression assay. In these separate detection experiments for GST and EGFR, we observed that the GST probe had a linear increase in Ct values between 0.13 and 4.17 nM of GST input, while the EGFR probe for A431 lysate was linear from 1.9 to 30 μg/ml (Additional file 1: Figure S1C and D).

Next we tested the ability of the 5′ Prox-Oligo-conjugated anti-GST antibody and 3′ Prox-Oligo-conjugated anti-EGFR antibody pair to detect an interaction between the GST-SH2 and EGFR in A431 lysate. Among multiple attempts performed to optimize the binding conditions, we found two distinct methods that provided a favorable signal-to-noise profile (Fig. 1b & Additional file 1: Figure S2). In Method 1, antibodies are premixed with target proteins prior to SH2 binding, while in Method 2, GST-SH2 domains and EGFR (lysate) are incubated prior to antibody binding (See Methods for more details). As the results from both methods were nearly equivalent, Method 1 (Fig. 1b) was used for all experiments described in this report.

### Specificity of the SH2-PLA

**Fig. 2:**
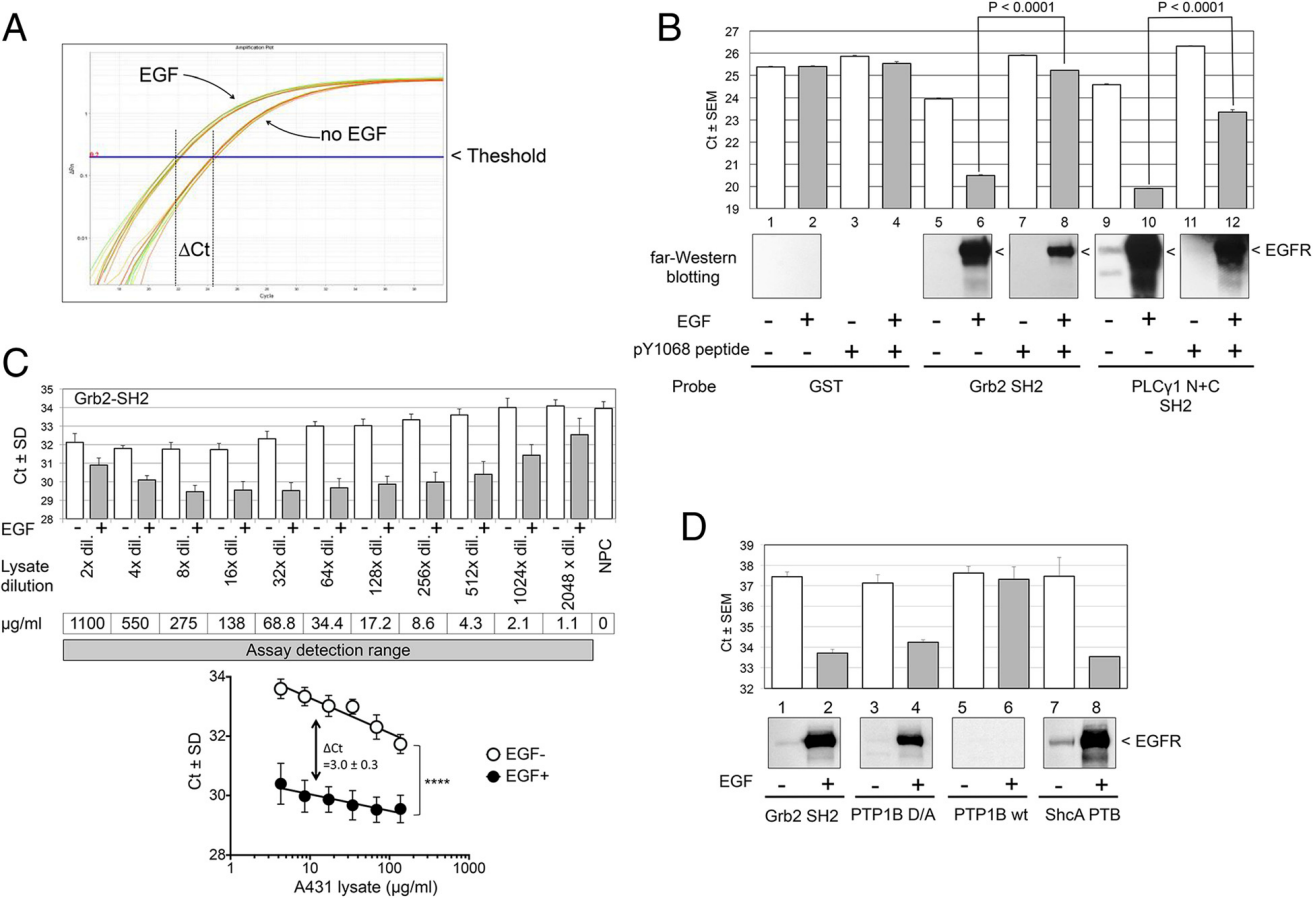
Validation of the SH2-PLA assay. **a** *,* Representative PCR amplification plot for SH2-PLA experiments. Increased binding between SH2 and pEGFR upon EGF stimulation is expressed as a reduced threshold cycle value (Ct). Here, ∆Ct is defined as [Ct_control_ – Ct_EGF stimulated_]_._ **b**, Specificity of SH2-PLA. SH2-PLA (top panel) and far-Western (middle panel) results for EGF-stimulated and control A431 cell samples are shown. Results for GST control probe are shown in lanes 1–4; Grb2 SH2 probe in lanes 5–8; and PLCγ1 tandem SH2 probe in lanes 9–12. Lanes 3, 4, 7, 8, 11, and 12 show the assay result in the presence of pY1068 blocking peptide, which contains the Grb2 SH2 consensus binding site of EGFR. **c** *,* SH2-PLA assay performance. The SH2-PLA assay was performed three times using a two fold dilution series of EGF-stimulated and control A431 cell lysates. Average Ct values, normalized to non protein control (NPC), are shown in the upper panel. The intra-assay variation for Ct values was 0.07-2.36 (mean 0.60) and the inter-assay %CV was 0.32-3.08 (mean 1.28). Since EGF-stimulated samples always showed a greater signal (lower Ct) than the unstimulated control throughout the dilution series, the range of assay detection is estimated to be at least 1.1–1100 μg/ml of lysate concentration, and the lower limit of detection is approximately 2 ng of protein per assay. The lower panel shows the approximately linear region of the mean Ct plot against log input lysate concentrations, and the ∆Ct (unstimulated – stimulated) of about three cycles. The log_2_ fold change between EGF-stimulated and control samples was estimated to be 6.0 - 6.4 using the ProteinAssist software tool (Additional file 1: Figure S3). **d**, Adoption of other phosphotyrosine recognizing domains. The SH2-PLA methodology was applied to protein tyrosine phosphatase (PTP) and phosphotyrosine binding (PTB) domains. ShcA PTB domain and the substrate-trapping mutant of PTP1B PTP domain displayed activity comparable to Grb2 SH2 (lanes 1–4 and 7–8). Signal was undetectable for the wild type (wt) PTP1B PTP domain, likely due to the intrinsic phosphatase activity (lanes 5–6)

A431 cell lysates were prepared in the presence or absence of EGF stimulation and the SH2-PLA assay was performed as outlined in Fig. 1b. We employed several SH2 domain containing proteins for validation that are known to be physiological ligands of EGFR such as Grb2, Vav2, and PLCγ1 [26–28]. Figure 2a shows a representative real-time PCR amplification plot of the SH2-PLA assay using the Grb2 SH2 domain probe and A431 cell samples. PCR product in the EGF-stimulated A431 sample was amplified more rapidly than in the unstimulated sample resulting in a lower threshold cycle (Ct) value. The difference in Ct values between the two samples (∆Ct) is an indicator of enhanced binding by the Grb2 SH2 domain probe to tyrosine phosphorylated EGFR (pEGFR). To validate the specificity of the assay, we compared signal from a GST-SH2 domain probe and GST control. The Ct value for the GST control was unchanged with EGF stimulation (lanes 1 vs. 2, Fig. 2b upper panel). On the other hand, the SH2 domains of Grb2 and PLCγ1 showed a marked reduction in their Ct values upon stimulation (lanes 5 vs. 6 and 9 vs. 10). Using the same set of samples and SH2 domains, far-Western blotting was performed as a reference (Fig. 2b middle panel). In far-Western, proteins were separated on polyacrylamide and transferred to a nitrocellulose membrane which was then probed with HRP-labeled GST-SH2 domains [23]. The identity of a major band at approximately 180 KDa in the SH2-far-Western blotting has previously been confirmed to be EGFR by anti-EGFR immunodepletion (data not shown). As shown in the middle panel of Fig. 2b, the signal profiles of the SH2-PLA and far-Western are similar despite their use of distinctive assay readouts (Ct values vs. bands). SH2 domains are known to have both unique and overlapping ligand binding characteristics [7, 29–32]. To determine if the SH2 binding is tyrosine site dependent, a synthesized phosphopeptide corresponding to EGFR tyrosine 1068, containing the Grb2 SH2 consensus binding site, was added as a blocker. In both assays, Grb2 SH2 binding was significantly reduced in the presence of the blocker, while the blocking effect on PLCγ SH2 domain binding was relatively modest (lanes 6 vs. 8 and 10 vs. 12). Taken together, these results indicate that, like far-Western, the SH2-PLA assay performed with the anti-GST 5′ Prox-Oligo antibody and anti-EGFR 3′ Prox-Oligo antibody probe pair is specific enough to distinguish between EGF-stimulated and control cell samples.

### Performance of the SH2-PLA assay

To evaluate assay performance, including the limit of detection, linearity, and precision, we performed the SH2-PLA assay using a serial dilution of lysate. In a 96 well plate, EGF-stimulated and control A431 cell lysates at concentrations between 1.1 and 1100 μg/ml were incubated with the Grb2 SH2 probe. Surprisingly, at all 11 lysate concentrations tested, EGF-stimulated samples showed a greater signal (lower Ct value) than unstimulated samples, demonstrating the high sensitivity of the system (limit of detection: ~1 μg/ml or 2 ng protein per assay). However, at higher lysate concentrations (>300 μg/ml), suppressed signal was observed (Fig. 2c upper panel), consistent with other reports using homogeneous proximity ligation assays [12, 19, 22]. This is often referred to as the “high-dose hook effect” and has been observed in other antibody based assays [33–35]. As a result, the assay had a linear signal response range of a 1–2 order of magnitude (Fig. 2c lower panel). In addition, the slopes of stimulated and unstimulated samples, an indicator of PCR amplification efficiency, were slightly different suggesting that conventional ∆∆Ct or standard curve methods are not suitable for relative quantification of SH2 binding. Therefore, we utilized ProteinAssist, a software tool designed for fold change estimation in TaqMan Protein Expression Assays based the ∆Ct squared method [12]. With this method, the log_2_ fold change between EGF-stimulated and control samples was estimated to be 6.0 - 6.4 (Additional file 1: Figure S3). Based on three independent assays, the intra-assay variation of ∆Ct values was 0.07-2.36 (mean 0.60) and the inter-assay %CV was 0.32-3.08 % (mean 1.28 %). Taken together, these results suggest that the limit of detection and precision are favorable, but the linear signal response range is modest, likely due to the binding characteristics of antibodies and probes.

### Application to other pTyr recognition domains

In addition to SH2 domains, members of the phosphotyrosine binding (PTB) and tyrosine phosphatase (PTP) domain families are also known to recognize phosphotyrosine residues and play regulatory roles in tyrosine kinase pathways [1, 36, 37]. Considering their potential applications in phosphoproteomics research, we tested if the same SH2-PLA methodology is applicable to these domains. GST-fusion proteins of the ShcA PTB domain, wild type PTP1B PTP domain, and catalytically inactive (substrate-trapping) mutant PTP1B PTP domain were purified and subjected to the assay using the same protocol as for SH2 domains. As shown in Fig. 2d, ShcA PTB and the catalytically inactive PTP domain of PTP1B showed binding activity to pEGFR comparable to the Grb2 SH2 domain, while the wild type PTP domain, having intrinsic PTP activity, showed no binding.

### Estimation of EGFR phosphotyrosines at the limit of detection

The serial dilution experiment indicated that the limit of detection is about 2 ng protein per assay in the case of A431 cells, although this threshold could change for other cell lines with different EGFR expression levels. A titration experiment using a “spike-in” pEGFR protein control would address the ambiguity, but preparation of such a reagent is challenging. Therefore, we took a retrospective approach in which a series of quantification methods were combined to measure the absolute amount of EGFR phosphotyrosine in the minimum amount of cell lysate necessary for SH2-PLA.

**Fig. 3:**
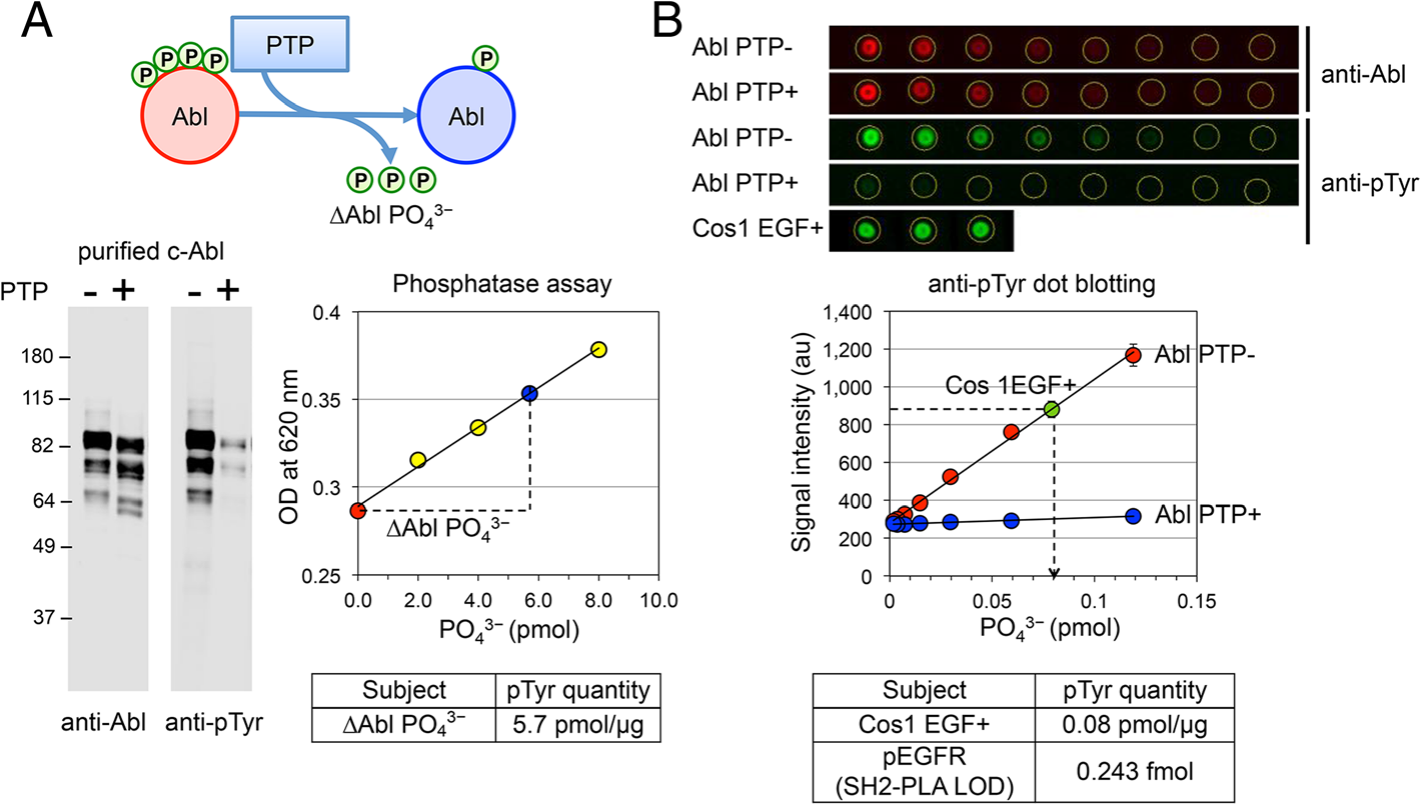
Estimation of EGFR phosphotyrosines at the limit of detection. To define an absolute lower limit of detection (LOD) for SH2-PLA, the total amount of EGFR phosphotyrosines in sample cell lysate was estimated using a phosphotyrosine standard sample and quantitative dot blotting analyses. **a**, Recombinant c-Abl protein, the pTyr-standard sample, was treated with tyrosine specific phosphatases PTP1B and TC-PTP. The amount of hydrolyzed phosphotyrosine was quantified by malachite green phosphatase assay (∆Abl PO_4_^3−^). Left panel shows anti-Abl and anti-phosphotyrosine blots for phosphatase-treated (Abl PTP+) and -untreated (Abl PTP-) samples. After the PTP treatment, the level of c-Abl tyrosine phosphorylation was greatly reduced but weak phosphorylation was still detectable with longer exposure time. The right panel shows a plot of the phosphate standard used for the quantification. Red circle, untreated c-Abl; blue circle, PTP-treated c-Abl; yellow circles, the kit supplied phosphate standard. From this analysis, ∆Abl PO_4_^3^ was estimated to be 5.7 pmol per μg of the c-Abl protein. **b** *,* Quantitative dot blotting. The total pTyr in the EGF-stimulated Cos1 cell lysate was estimated from a pTyr standard curve generated from anti-phosphotyrosine dot blotting. Upper panel shows raw anti-Abl and anti-pTyr blots. Serially diluted c-Abl pTyr standard (left to right 3.1–0.02 ng per spot) and 0.01 μg EGF-stimulated Cos1 samples were spotted on nitrocellulose membrane (performed in triplicate). The middle panel shows the resulting pTyr standard plot with the quantified signal intensities. The pTyr amount in the EGF-stimulated Cos1 lysate was estimated to be 0.08 pmol per μg lysate. Subsequently, an anti-pTyr Western analysis for A431 and Cos1 samples was performed, relative intensities of the EGFR bands were calculated, and the amount of EGFR pTyr in the EGF-stimulated A431 sample was estimated to be 0.122 pmol/μg. Thus 2 ng of EGF-stimulated A431 sample, which is the lower limit for SH2-PLA detection, would contain 0.243 femtomole EGFR pTyr. See Methods and Additional file 1: Figure S4 for more information

First, using a baculovirus expression system, we generated recombinant GST fused c-Abl protein to serve as the standard for phosphotyrosine. Then we treated the Abl protein with the tyrosine specific phosphatases PTP1B and TC-PTP. Anti-phosphotyrosine blots of treated and untreated Abl proteins indicated that phosphorylated Abl protein was mostly dephosphorylated by phosphatase treatment. Following the treatment, we quantified the amount of free phosphate, which is the hydrolyzed product of phosphotyrosine, using a phosphate standard curve generated with a malachite green phosphatase assay (∆Abl PO_4_^3−^ = 5.7 pmol/μg, Fig. 3a).

Next, anti-phosphotyrosine dot blotting was performed and the amount of total phosphotyrosine in EGF-stimulated Cos 1 lysate was estimated using the Abl standard (Fig. 3b). Finally, the tyrosine phosphorylation of EGFR was estimated by comparing the EGFR band intensity with the whole band intensity on an anti-phosphotyrosine Western blot (Additional file 1: Figure S4). According to these analyses, we estimated that 0.122 pmol phosphotyrosine is present on EGFR in 1 μg of EGF-stimulated A431 cell lysate. Thus in 2 ng lysate, which is the quantification limit of SH2-PLA (Fig. 2b), there is 0.243 fmol of phosphotyrosine on EGFR (Fig. 3b). In addition, assuming that A431 has 2.5 million EGFR per cell [38–40] and 150 pg of total protein per cell [41], we estimated that on average 4–5 tyrosines out of 22 putative tyrosine phosphorylated sites [42] per EGFR molecule are phosphorylated in EGF-stimulated A431 cells (Additional file 1: Figure S4).

### Practical limit of detection from cell culture

**Fig. 4:**
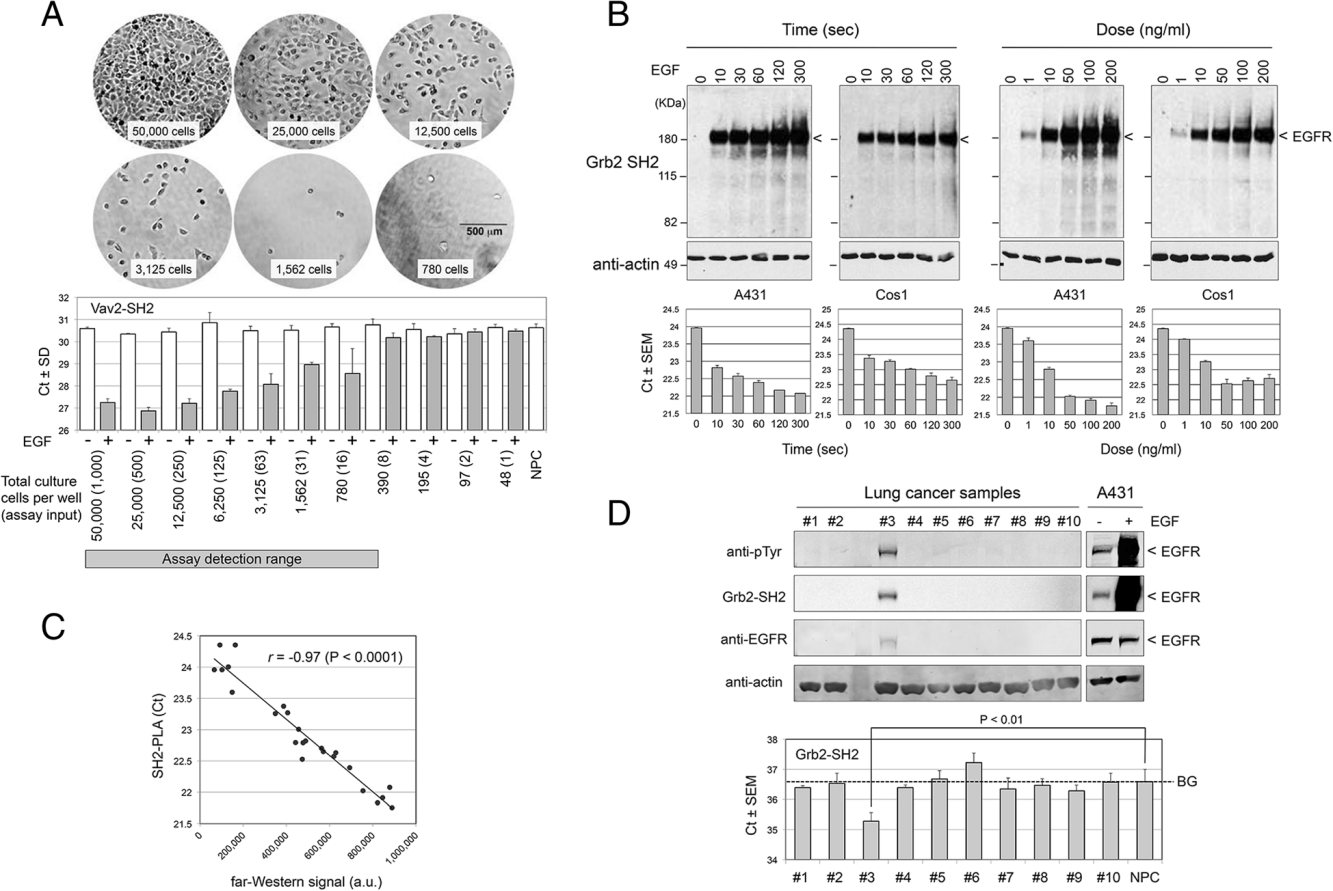
Applications of SH2-PLA assay. **a**, Practical limit of detection from cell culture. Two-fold serial dilutions of A431 cells were seeded to wells in a 96-well plate. Image series shows various 10x magnifications of diluted A431 cells with cell number indicated. Vav2 SH2:pEGFR interaction of starved or EGF-stimulated cell lysates was quantified by the SH2-PLA assay to resolve the assay detection limit. The Ct values from real-time PCR are shown with approximate numbers of cells per culture well or per assay (in brackets) underneath the chart. **b** *,* Time course and dose response of EGF stimulation. A431 and Cos1 cells were starved and stimulated with EGF at various times and concentrations as indicated. Upper panel shows far-Western blotting with Grb2 SH2 (25 μg lysate loaded per lane) and control blotting with anti-actin. Bottom panel shows Ct values of comparable SH2-PLA experiments loading 0.4 μg lysate per assay well. **c** *,* Correlation between far-Western and SH2-PLA assay. Using experimental results shown in *B*, EGFR band intensities of far-Western blot (X-axis) and average Ct values of SH2-PLA (Y-axis) in panel B were plotted and showed strong correlation. a.u., arbitrary unit; *r*, Pearson correlation coefficient. **d** *,* Application of SH2-PLA for cancer tissue analysis. The SH2-PLA/Western/far-Western analyses were performed using 10 lung cancer tissue samples. Upper panel shows Western and far-Western results with antibody/probe names indicated on the left. Only one sample (#3) shows an EGFR size band which also overlapped with bands detected by anti-pTyr and Grb2 SH2 (far-Western image represents 60-min exposure). The tyrosine phosphorylation level of the band is similar to the weak phosphorylation of EGFR in unstimulated A431 cells (right panel). The PLA-SH2 results for the same set of lung cancer samples are shown on the bottom. Consistent with the Grb2 far-Western result, only sample #3 had significant signal beyond the no protein control (NPC). The BG line indicates the background Ct value

Since the lysate dilution experiment indicated that the lysate requirement for SH2-PLA is very low, we next determined the lower limit of the assay by cell numbers. Serially diluted A431 cells were seeded in a 96-well plate, starved 16 h, and stimulated with EGF. Cells were lysed in the same volume of buffer and interaction between EGFR and Vav2 SH2 was analyzed by SH2-PLA. As shown in Fig. 4a, the assay detected EGF dependent SH2 interaction in the lysate equivalent of 16 cells (780 cells per well lysed in 50 μl) or approximately 2.5 ng which is close to the detection limit calculated above (Fig. 2c). In addition to EGFR-overexpressing A431 cells, we performed a similar lysate dilution experiment using EGF stimulated Cos1 cells and found that a lysate concentration approximately four times higher is required for detection, consistent with modest EGFR phosphorylation in Cos1 cells (Additional file 1: Figure S4 and data not shown). These results demonstrate that SH2-PLA is capable of detecting interaction between SH2 domains and pEGFR using a very small number of cultured cells.

### Correlation between SH2-PLA and far-Western in kinetic analyses

Defining phosphorylation kinetics in growth factor stimulated cells is important in cell signaling studies. We applied the SH2-PLA approach to determine time-dependent and dose-dependent changes in SH2 binding to pEGFR using A431 and Cos1 cells (Fig. 4b). For cross-validation, we performed far-Western analysis and compared the results side by side. As shown in Fig. 4b, the dose- and time-dependent increases in Grb2 SH2 binding to EGFR were obvious both by far-Western (~180 KDa band on upper panel) and SH2-PLA (Ct value in lower panel). When corresponding signal values (band intensity and Ct values) of both assays were compared, there was a high correlation (Pearson coefficient *r* = 0.97) (Fig. 4c). These results suggest that SH2-PLA is capable of analyzing cells with various levels of EGFR expression and is able to produce quantitative results similar to those obtained from established protein-protein interaction assays (*e.g.*, pull-down and immunoprecipitation) in a significantly shorter assay time while using 10–100 times less sample.

### Application of SH2-PLA for cancer tissue analysis

To explore the translational application of SH2-PLA, we analyzed binding of Grb2 SH2 to EGFR in lung cancer patient tissues. Empirically, the tyrosine phosphorylation analysis of patient-derived solid tumors is challenging due to intrinsically high protease and phosphatase activities requiring phospho-enrichment and larger starting material [43]. We asked if SH2-PLA is capable of detecting a specific signal within human lung cancer tissues without pTyr enrichment. In collaboration with the Haura group at the Moffit Cancer Center, we obtained 10 non-small cell lung cancer samples in a single-blind manner (neither molecular nor pathological characteristics were provided). We lysed the frozen tissues and performed the SH2-PLA assay (with the Grb2 SH2-anti EGFR probe pair), Western blotting (with anti-phosphotyrosine antibody), and far-Western blotting (with Grb2 SH2 probe) for comparison (Fig. 4d). In Western and far-Western, only one sample showed tyrosine phosphorylation at a band corresponding to EGFR size (#3, upper and middle panels). In the SH2-PLA assay using 0.5 μg of lysate per assay (equivalent to ~3,000 cells), we also observed a modest but significant signal (lower Ct value) in the same sample confirming the presence of tyrosine phosphorylated EGFR (lower panel), even though its level of phosphorylation was significantly lower than that of A431 cell samples (upper right panel). This result indicates that SH2-PLA is capable of detecting tyrosine phosphorylated SH2 domain recognition sites in weakly phosphorylated tumor tissues without requiring phosphopeptide enrichment.

## Discussion

In this study we developed SH2-PLA, a novel in-solution SH2 domain binding assay, to analyze tyrosine phosphorylated EGFR in EGF-stimulated cells. The key feature of this assay is that binding of the SH2 domain probe to its cognate phosphorylated binding sites is monitored in solution using a combination of proximity ligation and quantitative PCR technologies. A positive signal requires the formation of a quaternary protein complex, namely a 5′ oligonucleotide-tagged anti-GST antibody (anti-GST 5′ Prox-Oligo probe), a GST-SH2 protein, tyrosine phosphorylated EGFR, and a 3′ oligonucleotide-tagged anti-EGFR antibody (anti-EGFR 3′Prox-Oligo probe). We identified two stepwise protocols which yielded a suitable signal-to-noise profile: Method 1, pre-incubation of antibody probes to their respective targets (EGFR and GST) followed by SH2 binding; and Method 2, pre-incubation of the SH2 probe with EGFR followed by antibody binding. For EGF-stimulated A431 cells, we showed that a signal is detectable with as little as 2 ng lysate input per reaction and that the assay is able to detect specific interactions across three orders of magnitude of lysate input. To test the practical application, we conducted phosphorylation kinetics experiments of EGF-stimulated cells and observed good agreement between SH2-PLA and the established far-Western assay. Further we showed that SH2-PLA unambiguously detected a relatively low level of EGFR phosphorylation in lung cancer tissues using far less cell lysates than typically available from a core needle biopsy (~10^5^ cells or 100 μg) [44]. These data suggest that SH2-PLA is a promising platform to rapidly determine SH2 domain binding sites in EGF-stimulated cells or cancer tissues using a small amount of sample.

As with other immunoassays such as ELISA and bead arrays, various types of SH2 and other modular domain binding assays in both solid- and solution-phase platforms have been described [2, 45]. Among those, pull down, far-Western and their derivatives represent the most popular methods to assess SH2 domain binding sites on target proteins (*e.g.*, pEGFR). In pull down assays, lysate is incubated with bead-bound SH2 domains for several hours, followed by Western blotting with specific antibodies to the target protein. This assay requires a rigorous washing step and a negative control to minimize signal from non-specific binding and can be confounded by indirect binding via multi-protein complexes. In far-Western blotting, only direct binding between the labeled SH2 domain and membrane-bound proteins is detected, though the identity of the band may need to be confirmed by immunoprecipitation or other methods. As a result of these multiple procedures, these assays are usually low throughput, requiring 100–1000 μg of protein and multiple days of assay runtime. In contrast, SH2-PLA requires only a few microliters of lysate and 6–7 hours of assay time, making this assay highly flexible. Although other high throughput systems, based on microarray or fluorescence polarization, are suitable for global mapping of SH2 domain binding sites using synthesized peptide libraries [29, 32, 46, 47], SH2-PLA excels in the detection of SH2 binding to a specific target protein in whole cell lysate without requiring pre-purification or enrichment.

Although the assay sensitivity is high, we observed a relatively modest linear range (1–2 orders of magnitude). It is currently unknown that whether this can be attributed to biochemical characteristics of assay components including buffer solutions, oligonucleotides, antibodies, GST-SH2 domains, and the stoichiometry of tyrosine phosphorylation sites on EGFR. Consistent with our observation (Additional file 1: Figure S1A), the linear range for the TaqMan Protein assay system is reported to be 2–3 orders of magnitude when used to quantify protein expression [12, 20]. The anti-EGFR and GST antibodies used in this study when tested individually showed a lower linear range (Additional file 1: Figure S1C and D). In addition, the SH2-PLA, designed to detect the formation of the quaternary protein complex, may also be affected by the transient nature of SH2 domain-ligand binding [48–50]. Thorsen *et al.* have reported a feasibility study correlating the MMP9:TIMP1 protein complex with breast cancer prognosis in which they validated the use of ELISA and PLA-based methods [51]. In this report, they obtained a linear signal range across approximately one order of magnitude in human plasma dilutions (10X to 100X dilution). Taken together, multiple factors might result in the relatively narrow linear range seen in these protein-protein interaction assays employing in-solution proximity ligation assay. Nevertheless, this issue can be partly addressed by calibration using software tools such as ProteinAssist [12] (Additional file 1: Figure S3). Moreover, in many cases protein interaction assays are qualitative rather than quantitative in which the identification of positive interactions is the priority and high sensitivity and wide detection range, such as that observed in SH2-PLA, is essential.

Performance of SH2-PLA could be improved by modifying the methodology. Firstly, direct labeling of SH2 domains or the GST tag with oligonucleotides would reduce the assay complexity and could prevent issues stemming from insufficient GST:anti-GST antibody binding. Further, if different SH2 domains are tagged with different oligonucleotides, it would allow for a multiplex PLA reaction analogous to the previously described competitive reverse-phase SH2 profiling assay [8]. A potential problem with direct tagging is SH2 domain activity loss due to amino acid modification that would need to be assessed on a domain-by-domain basis. Secondly, solid-phase PLA could be considered. Solid-phase PLA uses an additional antibody which captures the target prior to dual recognition with 3′ and 5′ PLA antibody probes [14]. Solid-phase PLA was reported to have a broader dynamic range compared to solution-phase PLA [52, 53]. Owing to its additional washing steps, solid-phase PLA is likely less affected by the presence of excess unbound PLA probes and interfering substances in the buffer. 4-PLA, a more elaborate variation of solid-phase PLA, is another choice [54]. In 4-PLA, a set of five antibodies consisting of one capture antibody and four oligonucleotide-bound antibodies are employed to attain simultaneous recognition of five epitopes on one or more target molecules. Tavoosidana *et al.* applied 4-PLA to quantification of prostasome microvesicles, a potential marker of prostate malignancies, in blood samples and showed superior performance over a conventional PLA. A tempting application of this method would be a multiplex modular domain assay targeting multiple PTMs on receptor tyrosine kinases such as EGFR or PDGFR. Receptor PTMs could be monitored by a set of PTM-recognizing domains, *e.g.*, SH2, 14-3-3, *etc.*, allowing for identification of exclusive or simultaneous modifications. The lower background of these solid phase platforms could benefit SH2-PLA with improved overall sensitivity. However additional binding and washing steps will compromise the simplicity of the current SH2-PLA method. More importantly, it remains to be determined if weak SH2-pTyr interactions would be detectable after a stringent wash.

In addition to the SH2 domain, we demonstrated that this PLA-based methodology is a promising platform for the interrogation of other domain-based interactions. We showed that PTB and substrate-trapping PTP domains are capable of detecting EGFR phosphorylation. Although the SH2 domain is the most prevalent type of phosphotyrosine binding domain, integration of these other domains into the phosphorylation profiling is advantageous in that they allow for a larger coverage of tyrosine phosphorylation sites.

One obstacle to developing a customized SH2-PLA method for phosphorylated proteins will be preparation of a positive control. The original TaqMan Protein Assay is designed to quantify protein expression, making a purified protein target the most suitable control for determining assay performance. However, in the case of the SH2-PLA, a protein interaction assay assessing phosphorylation of a target protein, preparation of a suitable control is not a trivial task. For example, since SH2 domains may bind to a multiple tyrosine phosphorylation sites on the target protein, an appropriate positive control suitable for spike-in experiments should be phosphorylated on all SH2 binding sites and contain the antibody binding motif. We sidestepped this issue by establishing tyrosine phosphorylation standards and quantifying the absolute amount of tyrosine phosphorylation in the given sample, thereby estimating the assay detection limit. While this approach is applicable to any tyrosine phosphorylated protein target, similar approaches could also be tailored for other domains recognizing post-translational modification sites on cellular proteins [55]. This feature as well as the universality of GST-tagging could allow SH2-PLA to serve as a prototype for the development of powerful, convenient modular domain based proteomic tools [45].

## Conclusions

We have developed SH2-PLA, an alternative in-solution homogenous SH2 domain binding assay based on proximity ligation and real-time PCR. Using SH2-pEGFR interactions as the assay target, we showed that SH2-PLA has sufficient specificity, a very low limit of detection, a 1–2 order of magnitude linear range, and high reproducibility. In experiments with various EGF-stimulated cells, we confirmed good agreement between SH2-PLA and established far-Western assay results. Further, we found that SH2-PLA is sensitive enough to detect the relatively low level of pEGFR in lung cancer tissues without enrichment of pTyr-containing proteins. To our knowledge, this is the first report describing a microliter-scale assay for the detection of interaction between a recombinant SH2 domain probe and EGFR in cell lysate. This method provides significant improvement over traditional protein interaction assays requiring large sample input. With the low sample requirement and short assay runtime, SH2-PLA can provide a useful platform on which to develop high throughput modular domain binding assays applicable in both basic and translational cancer research.

## Methods

### Cell culture and sample preparation

Epidermoid carcinoma cell line A431 and monkey kidney fibroblast-like cell line Cos1 were maintained in DMEM supplemented with 10 % fetal bovine serum and 0.1 % penicillin/streptomycin (Mediatech). For EGF stimulation, overnight starved cells were stimulated with 50 ng/ml (0.37 nM) human recombinant Epidermal Growth Factor (EGF, Millipore Upstate) for 5 min and immediately lysed in Sample Lysis buffer (Applied Biosystems) with 1 mM phenyl methyl sulfonyl fluoride (PMSF), 1 mM sodium orthovanadate, and 5 μg/ml Aprotinin (Sigma A6279). For the cell titration experiment (Fig. 4a), two-fold serial dilutions ranging from 48 to 5 × 10^4^ A431 cells were seeded in a 96-well plate. Cells were starved for 16 h, stimulated with EGF, harvested, and lysed in 50 μl of the Cell Lysis Reagent (Applied Biosystems) with 1 mM PMSF, 1 mM sodium orthovanadate, and 5 μg/ml Aprotinin. Cleared lysates were used in SH2-PLA assay with the Vav2 SH2 domain. For time course and dose response experiments (Fig. 4b), cells were stimulated at different times (0–300 s) and doses (0–200 ng/ml). Immediately after stimulation, culture media was poured off and the dishes were snap frozen in liquid nitrogen. Frozen dishes were incubated with 300 μl Sample Lysis Buffer. Non small cell lung cancer tissues, originally banked at the Moffitt Cancer Center under its IRB protocol, were provided by Eric Haura (Moffitt Cancer Center) without clinical or biochemical information. The OCT-embedded frozen tissues were crushed, lysed in Sample Lysis Buffer, and solubilized using the Cryoprep and S2 homogenizer systems (Covaris). Cleared lysates were stored at −80 °C.

### GST fusion constructs

GST fusion proteins containing the Grb2 SH2 (amino acids 58–159), PLCγ1 SH2-SH2 (542–759), Vav2 SH2 (665–774), and ShcA PTB (127–317) were prepared as previously described [23]. Briefly, bacterial culture was incubated with 0.1 mM isopropyl β-D-1-thiogalactopyranoside (IPTG) and incubated at 16 °C for 24 h. After harvesting bacteria, proteins were affinity-purified on glutathione-Sepharose beads. Protein concentration was determined by Bradford assay (Bio-Rad), purity of protein was confirmed by SDS-PAGE, concentration was adjusted to 0.1 μg/μl with 10 % Glycerol-PBS, and stored at −80 °C. Similarly tyrosine phosphatase domain cDNA for human PTP1B (1–321, wild type and substrate trapping D181A mutant) and TC-PTP (1–354, wild type), both gifts from M.L. Tremblay, McGill University, were inserted into pGEX-6P1 (GE Healthcare), expressed in bacteria, purified, and stored at −80 °C.

### Western and far-Western blotting

25 μg (A431 and Cos1) or 40 μg (lung cancer tissues) of lysates were separated by SDS-PAGE and transferred to nitrocellulose membranes. Far-Western and Western blotting analyses were carried out as previously described [6, 7, 23]. Replica membranes were incubated with 200 nM GST fusion proteins in 5 % fat-free milk dissolved in TBST (150 mM NaCl, 10 mM Tris–HCl [pH 8.0], and 0.05 % Tween-20) for 2 h, washed for 20 min, and specific bands were visualized by chemiluminescence. Blots were stripped and reprobed with anti-EGFR (Santa Cruz Biotechnology), anti-phosphotyrosine (Cell Signaling), and anti-actin (Santa Cruz Biotechnology) antibodies. For quantitative far-Western analysis (Fig. 4b), band intensities were quantitated using ImageJ densitometry software (National Institutes of Health). For estimation of the absolute amount of tyrosine phosphorylation (Fig. 3 and Additional file 1: Figure S4), anti-phosphotyrosine Western blots were quantified using the LI-COR Odyssey IR detection system (LI-COR Biosciences).

### ICAM1 protein expression assay

Under the test site agreement, the TaqMan Protein Expression Assay kit was supplied from Applied Biosystems, part of Life Technologies. The supplied items included ICAM1 PLA assay probes; Raji B-cell lymphoma control lysate; Lysate Dilution Buffer; Assay Probe Dilution Buffer; Ab/Prox-Oligo Dilution Buffer; Assay Probe Storage Buffer; 5′ Prox-Oligo; 3′ Prox-Oligo; DNA ligase; Ligation Dilution Buffer; Ligation Reaction Buffer; protease; Universal PCR Assay; Fast Master Mix; Cell Lysis Reagent; Cell Resuspension Buffer; and an assay protocol. Additional reagents required for the assay were purchased from Applied Biosystems or other suppliers.

The Raji cell lysate (250 cell equivalent per μl) was serially 2-fold diluted in the Lysate Dilution Buffer in a 96-well plate on ice. 2 μl of 0.5 nM 3′ and 5′ ICAM1 assay probe mixture was added to 2 μl lysate in quadruplicate, sealed with MicroAmpTM Clear Adhesive Film, and incubated for 60 min at 37 °C. Subsequently 96 μl of DNA ligase in Ligation Dilution Buffer was added to the sample wells, resealed and incubated at 37 °C for 10 min to allow for the ligation reaction. The reaction was stopped by adding 2 μl of diluted protease solution to each well and the sealed plate was incubated at 37 °C for 10 min followed by heat inactivation at 95 °C for 5 min.

For real-time PCR reactions, 9 μl of the ligation product was mixed with 1 μl Universal PCR Assay solution and 10 μl of TaqMan Protein Expression Fast Master Mix in a real time PCR reaction plate. The ligation plate was sealed with MicroAmp Optical Adhesive Film, centrifuged at 1000 rpm for 10 s, and real-time PCR was performed using the StepOnePlus System with fast cycling condition (Applied Biosystems). The real-time PCR data including Ct values at threshold 0.2 with automatic baseline setting were exported from StepOne software and plotted by Ct values (cycle) and lysate input (cell equivalent per μl). Intra-assay variation was assessed using %CV of quadruplicates and inter-assay by Pearson correlation using Prism 6 software (GraphPad). For linearity assessment, Ct values were fitted by log-linear regression and R-squared values were calculated.

### Anti-GST and anti-EGFR probe development

Conjugation of biotinylated antibodies and oligonucleotide tags (5′ and 3′ Prox-Oligos) was carried out following the TaqMan Protein Expression Assay Probe Development Protocol (Applied Biosciences). To generate the anti-EGFR 3′ (or 5′) SH2-PLA probe, biotinylated anti-human EGFR antibody BAF231 (R&D Systems) was incubated with 3′ (or 5′) Prox-Oligo at 200 nM in Ab/Prox-Oligo Dilution Buffer at room temperature for 1 h with gentle rocking. The resulting 3′ anti-EGFR probe was diluted to 10 nM with Assay Probe Storage buffer and stored at −20 °C. The anti-GST 5′ (or 3′) SH2-PLA probe was prepared in the same manner using a biotinylated anti-GST antibody A00202 (Genscript) and 5′ (or 3′) Prox-Oligo.

To test the activity of the anti-GST 5′ and 3′ SH2-PLA probes, GST protein, purified and stored in −80 °C at 0.1 μg/μl in 10 % glycerol-PBS, was adjusted to 0.1 ng/μl with Cell Resuspension Buffer and 2-fold serially diluted in a 96-well plate on ice. The anti-GST 5′ and 3′ SH2-PLA probes were diluted in Assay Probe Dilution Buffer. The same volume of anti-GST 5′ and 3′ SH2-PLA probes were gently mixed, and 2 μl of mixed assay probes were added to 2 μl of serially diluted GST protein in duplicate (GST concentration: 0.13 nM – 4.17 nM). To test the activity of anti-EGFR SH2-PLA probes, protein lysates of 50 ng/ml EGF-stimulated and control A431 cells were adjusted to 0.03 μg/μl with Cell Resuspension Buffer and then 2-fold serially diluted in a 96-well plate on ice. 5′ and 3′ anti-EGFR Prox-Oligo probes were diluted in Assay Probe Dilution Buffer. The equal volumes of the 5′ and 3′ probes were gently mixed, and 2 μl of the mixture was added to 2 μl of serially diluted lysate in duplicate (final concentration of 0.93 – 30 μg/ml). Subsequent ligation reaction, protease treatment and real-time PCR were performed as described for the “ICAM1 protein expression assay.” In these experiments, the Ct value for unstimulated A431 lysate in a combination with anti-GST 5′ and anti-EGFR 3′ probes was used as a negative control (NC). The pY1068 blocker peptide for the Grb2 binding site on EGFR was purchased from Cell Signaling.

### SH2-PLA assay

Method 1: Lysate dilutions in Cell Resuspension Buffer were incubated with anti-EGFR 3′ PLA Prox-Oligo probe (final concentration 0.5 nM) for 1.5 h at 4 °C [Solution 1]. GST-SH2 protein was incubated with anti-GST 5′ Prox-Oligo probe (final concentration 0.1 μg/ml and 0.5 nM, respectively) for 1.5 h at 4 °C [Solution 2]. Then 2 μl of the Solution 1 and Solution 2 were incubated at 4 °C for 1.5 h in the thermal cycler to induce a quaternary complex of [anti-EGFR 3′Prox-Oligo probe:phosphorylated EGFR:GST-SH2 protein:anti-GST 5′ Prox-Oligo probe] (Fig. 1b). Subsequent ligation reaction, protease treatment and real-time PCR were performed as described for the “ICAM1 protein expression assay.”

Method 2: GST-SH2 or GST control proteins were diluted to 0.1 μg/ml in Cell Resuspension Buffer and incubated with the same volume of EGF-stimulated or unstimulated cell lysate for 1.5 h at 4 °C (for SH2-EGFR binding). Then 2 μl of the SH2 bound lysates were incubated with 2 μl of 0.5 nM Prox-Oligo probe mixture (anti-GST 5′ + anti-EGFR 3′) in a thermal cycler at 4 °C for 1.5 h to form a quaternary complex of [anti-EGFR 3′Prox-Oligo probe:phosphorylated EGFR:GST-SH2 protein:anti-GST 5′ Prox-Oligo probe] (Additional file 1: Figure S2). Subsequent ligation reaction, protease treatment and real-time PCR were performed as described in “ICAM1 protein expression assay.”

### Quantification of EGFR phosphotyrosines

A GST fusion of wild-type murine type IV c-Abl was produced from baculovirus-infected Sf9 insect cells as described previously [24]. GST-Abl was purified by incubating lysates with glutathione-agarose beads (GE) at 4 °C for 1.5 h, washing extensively in Buffer E (50 mM Tris pH 8.0, 150 mM NaCl, 5 % glycerol, 2 mM dithiothreitol) and eluting with Buffer E plus 20 mM reduced glutathione. Prior to the malachite green free phosphate assay, the GST-Abl solution was concentrated to about 0.5 μg/μl with an Amicon Ultra-4 Centrifugal Filter (10 K, Millipore).

Malachite green phosphate assays were performed according to the manufacturer’s instructions (Abnova). Briefly, purified GST-Abl and PTP cocktail (GST-PTP1B and GST-TcPTP) proteins are incubated at a 5:1 molar ratio at 37 °C for 2 h, then mixed with acidic solution for quenching. Serially diluted reactions were visualized with 1 mM malachite green and quantified by absorbance at 620 nM using the M1000 Pro plate reader (Tecan). A standard curve for the absorbance and phosphate input (PO_4_^3−^) was obtained by adding a serially diluted free phosphate standard into the untreated c-Abl solution. The change in phosphotyrosine (as assessed by PO_4_^3−^ release) in the c-Abl solution during the PTP treatment (∆Abl PO_4_^3−^) was estimated with the free phosphate standard curve (Fig. 3a).

For comparative dot blotting, Abl proteins and Cos1 lysates were diluted in SDS sample buffer, boiled, and manually spotted in triplicates on a nitrocellulose membrane (0.003 to 100 ng in 0.5 μl per spot). The dried membrane was incubated in Buffer T (10 mM CAPS pH 11.0, 20 % methanol) for 30 min, blocked in 5 % nonfat milk-TBST for 1 h, and incubated with anti-phosphotyrosine antibody (PY100, Cell Signaling) overnight at 4 °C. After washing with TBST (10 mM Tris pH8.0, 150 mM NaCl, 0.05 % Tween 20), membranes were incubated with IRdye conjugated goat anti-mouse antibody, washed, and bound antibody was detected using an infrared imaging system (Odyssey, LI-COR Bioscience). Raw signal of individual dots was quantified using Odyssey 3.03 and ImageStudio software (LI-COR Biosciences) and plotted against protein quantity (Fig. 3b).

The absolute amount of phosphotyrosine in the Cos1 sample, was estimated by the linear standard curve generated by serially diluting the Abl standard (Fig. 3b). Of note, although a trace amount of tyrosine phosphorylation is detectable in PTP-treated Abl, at low concentrations (3.1–0.02 ng per spot) the untreated Abl spots had a linear signal profile and were unaffected by the trace PTP-treated Abl signal. Thus the pTyr amount of the target sample can be interpolated using the linear Abl calibration curve (Fig. 3b). For EGFR band quantification, 10 μg of A431 and Cos1 cell samples were separated on SDS-PAGE, transferred to nitrocellulose in Buffer T, and immunoblotted with anti-phosphotyrosine antibody as described above. For EGFR band analysis, the mean values of whole lanes of EGF-stimulated samples were compared to EGFR size bands in Cos1 cells, which were then used to approximate the amount of phosphotyrosine on EGFR in A431 cells (Additional file 1: Figure S4).

### Data analysis and statistical tests

To evaluate assay performance, the Ct values were plotted against the lysate concentration (μg/ml) or cell number (cell per μl) and compared with those of unstimulated control samples. The linear signal range was estimated using Microsoft Excel 2011 (Microsoft), Prism 6.0 (GraphPad), and ProteinAssist (Applied Biosystems) as appropriate. Outliers were excluded by the ROUT method in Prism 6.0 with Q = 1.0 % [25]. The intra-assay variation was determined by %CV of Ct values from three technical replicates. Assay to assay (inter-assay) variability was assessed with normalized Ct values from three independent experiments, in which antibody binding, SH2 binding, ligation, protease treatment, and real-time PCR reactions were independently performed while using the same batch of cell lysate samples.

For fold change estimation, ProteinAssist v1.1 (Applied Biosystems), a software tool customized for TaqMan Protein Assays based on the ∆Ct squared method, was used [12]. Relative quantification was performed using serial dilution data for EGF-stimulated and control samples (Fig. 2 and Additional file 1: Figure S3). Raw Ct values were directly imported into ProteinAssist which generated the ∆Ct-by-input quantity plots. The quantification threshold, outlier detection, and linear range detection were set to default values and the automatically assigned linear range was manually corrected. The log_2_ fold change with 95 % confidential intervals between the stimulated and unstimulated samples for Grb2 SH2 binding was estimated by comparing each X-intercept (at quantification threshold set at 2.0) of the linear regions of the ∆Ct plots for the stimulated and unstimulated samples.

For clinical samples, the statistical significance of sample Ct value differences were assessed using an ordinary one-way ANOVA test with Dunnett’s multiple comparisons test using Prism 6.0.

## Additional file

Additional file 1: Figure S1. Performance of homogeneous in-solution PLA. **Figure S2.** Experimental workflow for SH2-PLA Method 2. **Figure S3.** Fold change calculation. **Figure S4.** SH2-PLA limit of detection estimation.

## Abbreviations

SH2: Src homology 2
PLA: Proximity ligation assay
EGFR: Epidermal growth factor receptor
pEGFR: Tyrosine phosphorylated EGFR
GST: Glutathione S-transferase
pTyr: Phosphotyrosine
PTB: Phosphotyrosine binding
PTP: Protein tyrosine phosphatase
PCR: Polymerase chain reaction
MS: Mass spectrometry
