# Supplementary material for: SH2-PLA: a sensitive in-solution approach for quantification of modular domain binding by proximity ligation and real-time PCR
Source: BMC Biotechnol. 2015 Jun 26;15:60. doi: 10.1186/s12896-015-0169-1 (PMC4482279; doi:10.1186/s12896-015-0169-1)
Supplement: Additional file 1: Figure S1. — Performance of homogeneous in-solution PLA. Figure S2. Experimental workflow for SH2-PLA Method 2. Figure S3. Fold change calculation. Figure S4. SH2-PLA limit of detection estimation. [file 12896_2015_169_MOESM1_ESM.pdf]

SH2-PLA: A Sensitive In-Solution Approach for Quantification of Modular Domain Binding by Proximity Ligation and Real-Time PCR. Thompson et al.

Additional file 1: Includes Figures S1-S4.

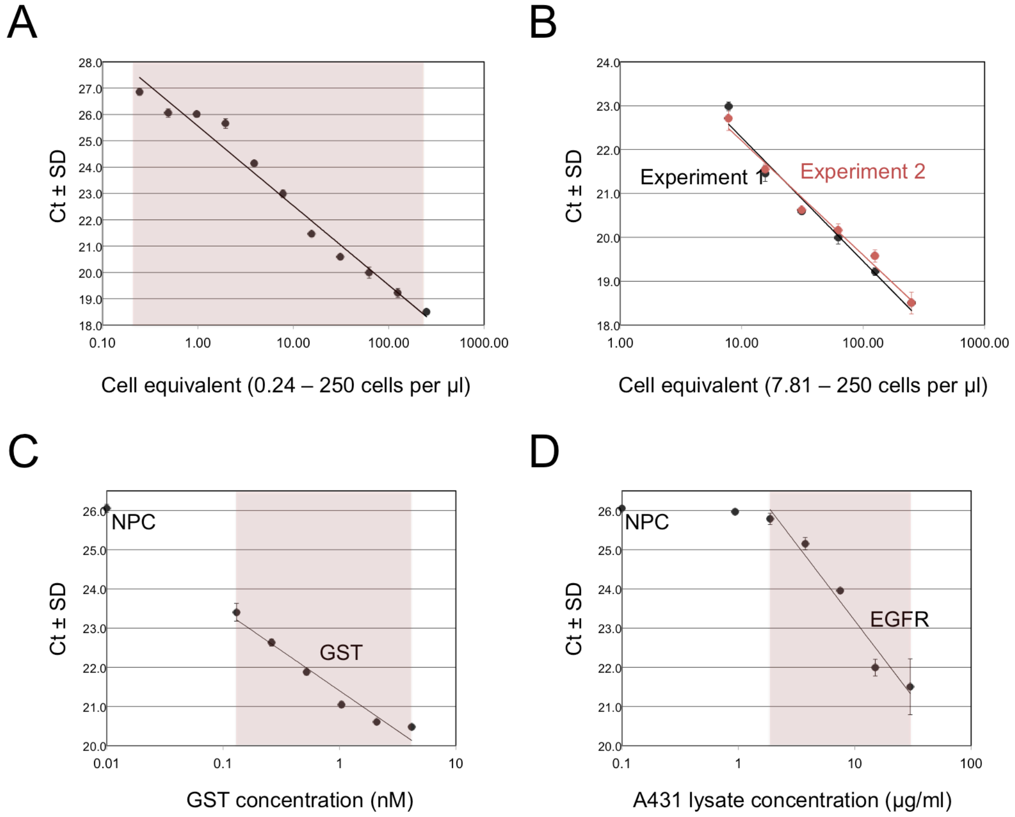

Figure S1. Performance of homogeneous in-solution PLA. TaqMan Protein Expression Assay was performed using the control Raji cell lysate and anti-ICAM1 antibody probes (*A and B*). *A,* The mean Ct plot against log input concentration (expressed as the number of cell equivalents) suggests at least three orders of linear dynamic range indicated by shade (0.24 – 250 cells per well). Intra-assay variation based on %CV from quadruplicates was 0.25 – 1.06%. *B,* Inter-assay %CV based on normalized Ct values from two independent experiments was 0.1-1.3%, Pearson correlation (*r* = 0.99). *C,* TaqMan Protein Expression Assay was performed using custom-made 3' and 5' Prox-Oligo anti-GST antibody probes. Ct values from real-time PCR were plotted against GST protein input (nM). The linear dynamic range for GST protein concentration fell between 0.13 and 4.17 nM (R^2 = 0.96). *D,* Using the 3' and 5' Prox-Oligo anti-EGFR antibody probe pair, the TaqMan assay was performed to detect endogenous EGFR in A431 cell lysate. Ct values from real-time PCR were plotted against A431 lysate input (µg/ml). The linear range fell between 1.9 and 30 µg/ml of the lysate (R^2 = 0.96). Shaded area indicates the linear range. Ct value for negative lysate control (NPC) is shown on the left.

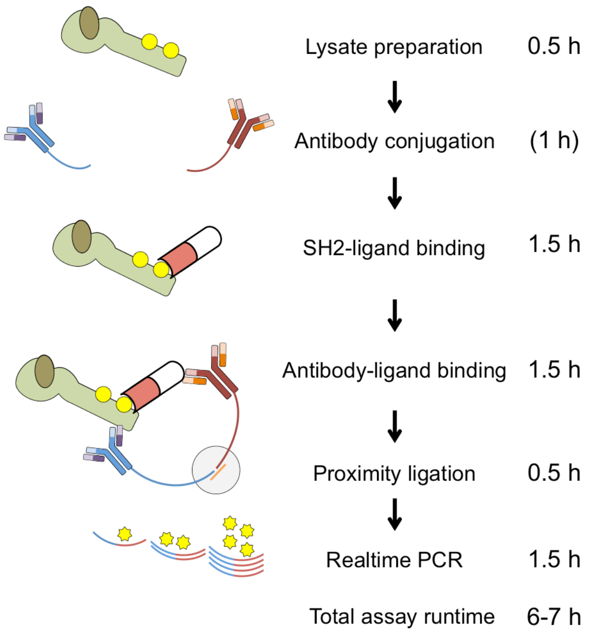

Figure S2. Experimental workflow for SH2-PLA Method 2. Lysates are prepared with or without EGF stimulation. Biotinylated anti-GST and anti-EGFR antibodies are conjugated with the 5’ and 3’ Prox-Oligos, respectively, and stored at −20˚C. The GST-SH2 is incubated with cell lysates for 1.5 h allowing the SH2 to bind EGFR in the lysate. Subsequently, the lysate-SH2 mix and both the 5’ and 3’ SH2-PLA probes are mixed to allow antibody-epitope binding. Then, the final complex is quantified by proximity ligation and real-time PCR. Estimated assay runtime including sample-handling steps for each procedure is noted on the right.

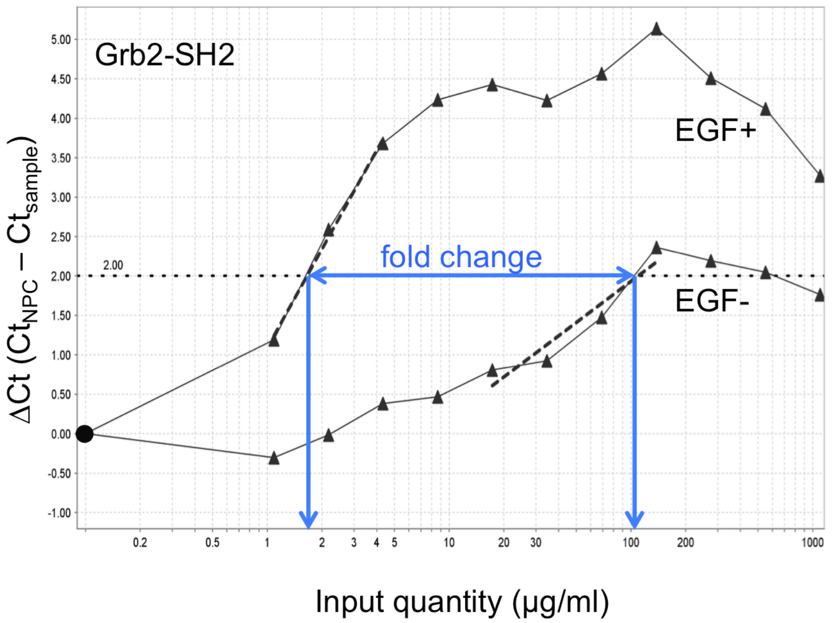

Figure S3. Fold change calculation. Using serially diluted EGF-stimulated A431 cell lysates (EGF+) and unstimulated control samples (EGF-), SH2-PLA experiments were performed three times. ΔCt values, which are the difference between sample and non protein control (NPC) Ct values, were plotted against input lysate quantity (1.1 – 1100 µg/ml). The fold change between the stimulated and unstimulated samples for Grb2 SH2 binding was estimated by comparing each X-intercept (at quantification threshold) of the linear regions of the ΔCt plots for the stimulated and unstimulated samples. X-intercepts were calculated using ProteinAssist software (Applied Biosystems) with the quantification threshold set at 2.00. Linear range estimation, quantification threshold, and outlier detection were set automatically with manual correction. A $\log_2$ fold change between 6.0 and 6.4 was obtained from the three independent experiments.

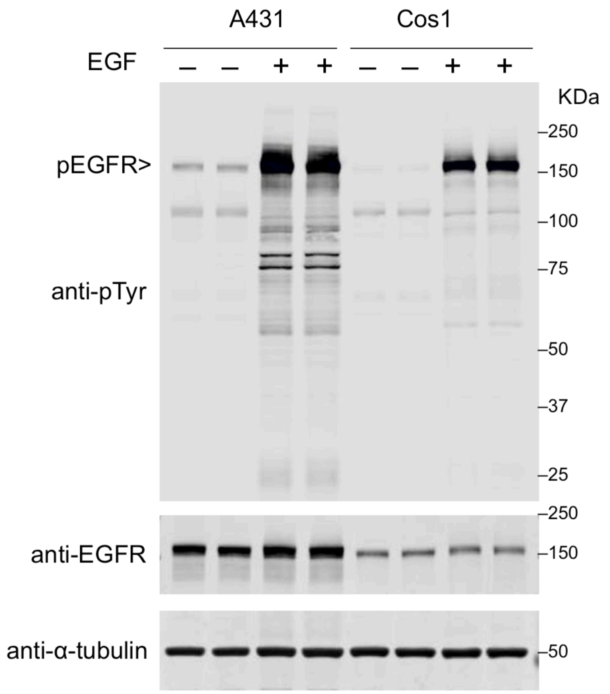

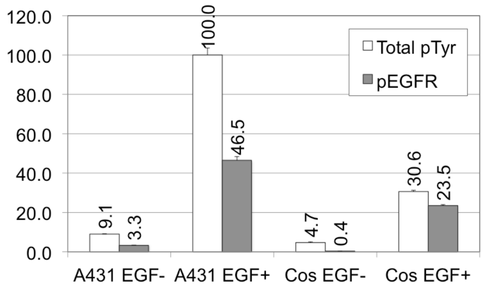

Subject	pTyr quantity
Cos1 EGF- total	0.012 pmol/μg
Cos1 EGF+ total	0.080 pmol/μg
Cos1 EGF+ pEGFR	0.061 fmol/μg
A431 EGF- total	0.024 pmol/μg
A431 EGF+ total	0.261 pmol/μg
A431 EGF+ pEGFR	0.122 pmol/μg
A431 EGF+ pEGFR	0.243 fmol/2 ng
pEGFR (PLA LOD)	0.243 fmol
pEGFR	4.39 phosphates per EGFR molecule

Figure S4. SH2-PLA limit of detection estimation. An equal amount of EGF-stimulated and control A431 and Cos1 lysates were subjected to Western analysis with anti-pTyr, anti-EGFR, and anti-α-tubulin antibodies using the LI-COR Odyssey IR detection system. Whole sample lanes and pEGFR-sized bands from the pTyr blot were quantified using LI-COR Odyssey IR imaging software. On the right graph, numbers indicate quantified pTyr signals normalized to the whole lane intensity of EGF-stimulated A431 cells. The absolute amount of phosphotyrosines on pEGFR in A431 and Cos1 samples were calculated by comparing relative intensities of bands with the total phosphotyrosine in Cos 1 (0.080 pmol/μg) (see Figure 3). Based on 1) the limit of detection value from the lysate dilution experiment (LOD: 2 ng A431 EGF+), 2) the total amount of pTyr in Cos1 (0.080 pmol/μg), and 3) the EGFR band ratio determined by this analysis, we estimated the SH2-PLA limit of detection to be 0.243 fmol of pEGFR phosphotyrosine. In addition, based on the assumption that A431 has 150 pg of total protein and 2.5 million EGFR per cell, the number of phosphates per EGFR in EGF-stimulated A431 was also estimated to be 4.39. Table on the right shows estimated pTyr values.
